# Perceptions of everyday life during lenient COVID-19 restrictions in Sweden- an interview study

**DOI:** 10.1186/s12889-023-16599-3

**Published:** 2023-09-07

**Authors:** Ingrid Lindgren, Anna Trulsson Schouenborg, Caroline Larsson, Kjerstin Stigmar

**Affiliations:** 1 Rehabilitation and Sustainable Health, Department of Health Sciences, Lund University, Lund, Sweden; 2 Department of Neurology, Rehabilitation Medicine, Memory Disorders and Geriatrics, Skåne University Hospital, Lund, Sweden; 3 Department of Neurosurgery and Pain Rehabilitation, Skåne University Hospital, Lund, Sweden; 4 Human Movement: health and rehabilitation, Department of Health Sciences, Lund University, Lund, Sweden

**Keywords:** Corona pandemic, Health, Well-being, Qualitative study, Restrictions

## Abstract

**Background:**

Both COVID-19 and its associated societal restrictions have affected individuals’ health and everyday life. In Sweden, more lenient public health restrictions were implemented, with individuals asked to act responsibly in terms of reducing spread of disease. The majority of studies reporting on experiences of Covid-19 restrictions have been in the context of more substantial mandatory rules aimed at reducing social contact, therefore it is important to describe how more lenient restrictions have impacted individuals’ well-being. This study aims to describe perceptions of everyday life during the first wave of the COVID-19 pandemic, perceived by individuals with no underlying medical condition, and living with more lenient public health restrictions in Sweden.

**Method:**

The participants were recruited from individuals who originally had participated in an online survey about life satisfaction, health, and physical activity. Fifteen individuals (median age 49, range 26–76 years, seven women) in various social situations, such as living alone/cohabiting, having children at home, geographical area and size of city were interviewed. Qualitative content analysis was applied to the data.

**Results:**

An overall theme “Both hindrances and opportunities in important life domains were experienced within the same person during lenient Covid-19 restrictions” was derived and covered three categories: “New possibilities of flexibility in work and better health”, “Life went on as usual with minor adjustments” and “Everyday life changed and became more difficult” together with eight subcategories. For most participants, both facilitating and hindering important domains in life were described. Unexpected findings were positive experiences regarding working from home, physical activities, leisure time activities and the balance between work and leisure time. In areas where only minor adjustments were made, life was perceived as going on as before. On the other hand, restrictions increased worries and were perceived to have negative effects on social participation.

**Conclusion:**

The impact of the pandemic and lenient restrictions in Sweden on the participants´ everyday life was multifaceted. Both hindrances and opportunities in important life domains were experienced within the same person. An increased flexibility in work- and leisure activities were perceived having positive effects for health and wellbeing and led to a better balance in life.

**Supplementary Information:**

The online version contains supplementary material available at 10.1186/s12889-023-16599-3.

## Background

The COVID-19 pandemic has caused enormous human suffering, challenged the foundations of societal well-being and profoundly changed our lives. Society and health care organisations have faced challenges never encountered before. In May 2022, the World Health Organization, WHO, stated that the number of cases reported globally had exceeded 500 million cases with an estimated over six million deaths [1]. By May 2022, Sweden reported over 18 000 Covid related deaths [1, 2]. The COVID-19 pandemic has over time emerged in waves and has impacted global regions to various extents. Health care has had a heavy burden managing patients with severe symptoms and death, but also managing patients with other types of diseases along with on-going COVID-19 infection.

In general, the pandemic has had a profound impact on individuals´ health and life [3–5]. But also, the restrictions and limitations introduced by authorities worldwide to slow the transmission of the virus have changed life routines in a perceptible way for many people. In comparison to many other countries, the Swedish policy has been less stringent [6]. The Swedish strategy has been based on voluntary restrictions and recommendations and no legal sanctions against individuals have been imposed [7]. Shops, theatres, sports arenas, and public areas have been obligated to follow activity-specific restrictions, concerning for example distancing and maximum number of attendances. However, also in Sweden the recommendations have implied social distancing, working from home and distance education at high school and universities. Sport activities for children and adolescents have been cancelled. Throughout the pandemic, these restrictions have been continuously adjusted. The Swedish government, and the Public Health Agency of Sweden have worked closely together with the ambition to alleviate the health care burden [8].

A growing body of research shows profound effects on wellbeing and health among people during the COVID-19 pandemic. In a review of the impact of quarantine, the authors included studies from North America, Africa, Asia, and Sweden. They concluded that an imposed quarantine, although necessary, may have long-lasting consequences and that it is important to minimize the impact of a quarantine by providing information and support [10]. Also, current literature suggests that psychological symptoms such as fear, anxiety, depression, stress, sleep disorders and suicidal behaviour were triggered by the pandemic itself as well as by the restrictions related to the pandemic [11, 12]. This is especially prevalent among people with pre-existing medical conditions [13].

Also, qualitative data is emerging regarding the perceptions and experiences of social distancing and social isolation. Most of these are based on the experiences of people suffering from underlying medical conditions such as long-time respiratory conditions [14], dementia [15], among pregnant women [16], older adults [17] or among children and adolescents [18]. However, a UK study based on public perceptions of individuals reporting no underlying medical conditions, revealed that many participants felt that social distancing and isolation policies have had significant negative social and psychological impacts on their lives [12]. Lack of trust in, and clarity of, government communication as well as high levels of self-adherence, but even observations of non-adherence in others were also reported [12].

Another study explored COVID-19 related to social stigma (meaning negative societal beliefs and feelings associated with the health status among confirmed coronavirus individuals and their household members) and the experiences of people who were home quarantined or isolated in Finland during the spring 2020 [19]. This study reported that social stigma could be a challenge by impacting social relations and by creating psychological distress, which is likely to affect quality of life. Experiences of quarantine and isolation were especially characterized by health worries and boredom.

Also, attitudes and beliefs among community members have been studied. The findings showed that the participants felt overwhelmed by staying at home and that they experienced frustration, agitation/anxiety, boredom, and loneliness due to lack of physical interaction [20]. Moreover, perceptions of shock and chaos, gradual adjustment to the new reality, fears, and concerns for oneself and family members have been described [9].

Thus, many studies have been conducted in a context of strict confinements and lockdowns. They indicate that this can have a huge impact on society in different ways. Governments and health care authorities make decisions on the best available facts and consider pros and cons for different interventions. In Sweden, the use of more lenient restrictions was applied, and this strategy has received criticism from other countries. To increase knowledge of the perceptions of more lenient restrictions and confinements, a large online survey was carried out in Sweden in the fall of 2020 [21, 22]. Somewhat surprisingly, a significant part of the participants reported an unchanged or improved level of physical activity and life satisfaction over the period. The likelihood of reporting decreased life satisfaction increased for those who had no children living at home; were middle-aged; did not receive their income from employment or had a chronic illness. Older age was also associated with reduced physical activity levels due to the restrictions. To further increase knowledge, this study aims to describe perceptions of everyday life during the first wave of the COVID-19 pandemic, perceived by individuals with no underlying medical condition, and living with more lenient public health restrictions in Sweden.

## Methods

The present study is part of a larger project on COVID-19, which encompasses online surveys on physical activity and life satisfaction during the COVID-19 pandemic [21, 22], and interview studies. We used the Consolidated Criteria for Reporting Qualitative Research, COREQ checklist [23] as a foundation for reporting the study. All four authors were engaged in all phases of this study, including recruitment, conducting interviews, and doing the analysis as well as manuscript writing.

### Recruitment of participants

Participants were recruited from a Facebook announcement from the Department of Health Sciences, Lund University, posted between 1^st^ of September and 7^th^ of October 2020. The announcement was also shared via Instagram and Twitter and comprised information about the project. The project webpage was hosted at Lund University.se and contained general information on the project, a participant information sheet, and a link to the survey. The announcement was directed to three different regions in Sweden: two regions with large outbreaks in the first wave of the pandemic (Stockholm, the capital of Sweden with approximately one million inhabitants, and Gothenburg, the second largest city with 600 000 inhabitants 2020), and to the county of Scania in the southernmost part of Sweden (approximately 1,4 million inhabitants 2020) consisting of rural areas, smaller towns, and medium-sized cities. Individuals aged 18 years or older, able to read and understand Swedish were invited to participate in the study. The announcement was also possible to share. Participants received information about the study, including the online survey and a potential interview. Informed consent to publish was obtained by each participant. In the online survey, 1082 people replied. The online survey was anonymous (i.e., included no names nor personal identification numbers), but included demographic questions and questions about life satisfaction, health, and physical activity [21, 22].

The participants in the online survey were asked to participate in an interview on perceived physical and mental wellbeing during the pandemic and if willing to do so, asked to provide their phone number. No names were registered. After the online survey was closed, the researchers sorted the participants who had provided their phone number (*n* = 277) depending on whether they had an underlying illness/ medical condition or not. In the present study, focus was on participants reporting no underlying medical condition. The potential participants (*n* = 159) were sorted by sex (76,4% women) and then into five age groups (< 35, 35–49, 50–69, ≥ 70 years). Each participant was labelled with the following: Sex, living alone or with a partner (yes/no); having children living at home or not (yes/no) and living in a large, medium, or small town. A table with groups based on these properties was formed. Our intention was to get a diverse sample regarding sociodemographic factors and using the table, we did a selection from each age group, representing both men and women and reflecting the different living situations [24, 25]. Fifteen potential participants were contacted by telephone consecutively. If the person did not answer at the first call, they were called a second time. All the 15 potential participants we got in contact with agreed to participate. During a first telephone contact, the researchers informed the participant about the study and ethical principles and booked an appointment for the interview. They were also told that they could interrupt the interview at any time. At the time for the interviews, one person was not able to be reached, therefore the next similar person on the list was contacted. Consequently, 15 persons agreed to be interviewed. All background characteristics were obtained from the online survey [21, 22] (Table 1).

**Table 1:** Description of study sample (*n* = 15)

**Participant number**	**Sex**	**Age group**	**Residential community size**	**Civil status**	**Education**	**Income**
1	Female	< 35	Small village	Married/cohabiting	Post-secondary education	Work
2	Female	35–49	Large City	Single	Post-secondary education	Work
3	Female	50–69	Medium-size town	Married/cohabiting	Post-secondary education	Work
4	Female	70 +	Large City	Single	Post-secondary education	Other
5	Male	35–49	Medium-size town	Single	Post-secondary education	Work
6	Male	35–49	Medium-size town	Single	Post-secondary education	Work
7	Male	50–69	Medium-size town	Single	Secondary school	Work
8	Male	< 35	Large City	Single	Secondary school	Studies
9	Male	50–69	Medium-size town	Married/cohabiting	Post-secondary education	Work
10	Male	< 35	Large City	Married/cohabiting	Secondary school	Work
11	Male	60–69	Large City	Married/cohabiting	Post-secondary education	Work
12	Male	70 +	Small village	Married/cohabiting	Secondary school	Other
13	Female	< 35	Medium-size town	Single	Post-secondary education	Work
14	Female	35–49	Small village	Married/cohabiting	Secondary school	Work
15	Female	70 +	Small village	Single	Unknown	Other

### Interviews

An interview guide with open-ended questions was developed in advance [26]. All four authors were involved, and the interview guide was adjusted several times. The questions focused on the participant’s everyday life situation during the pandemic and if there were any changes since the start of the pandemic. Questions were related to work situation, daily activities and leisure activities, social contacts, and physical and mental health (Additional file 1). The interview guide was tested on a person not involved in the project which rendered further minor adjustments.

The researchers (IL, CL, ATS, KS) who did the interviews had no relationship whatsoever to the participants. All interviewers were registered physiotherapists, educated to doctorate level and had long clinical and research experience (> 10 years). Two of the interviewers had also extended experience in doing interviews and qualitative studies. The interviews were conducted on telephone (13 participants) or a digital communication platform (Zoom) (2 participants) in a confidential way (i.e., names or city of residence were not mentioned) and recorded. It was mandatory to use the Zoom camera. The ambition was to have a conversation, so questions were asked, based on the interview guide, but not necessarily in the same order [27]. Probing questions were used to deepen interesting topics. The interviews took 21–54 min (mean 39 min).

The interviews were transcribed verbatim by two students, employed by the project. The audio recordings and the transcribed interviews were stored on a secure data server at Lund University, to which only the researchers and the persons who transcribed the interviews had access. All transcribed material was in Swedish, until manuscript writing. The quotes were forward translated by a bilingual speaking researcher with English as first language. This person also holds experiences in the area.

### Analysis

Data were analyzed applying latent qualitative content analysis [28, 29]. First, all the transcribed interviews were read by all authors several times, to get an overview and sense of the whole content. All transcribed text was considered as unit of analysis. Thereafter, one interview was analyzed jointly by all authors. The text from the interview was divided into meaning units, condensed, and labelled with codes by each author and was then discussed thoroughly. Then the remaining interviews were divided between (KJ, IL, ATS) for identification of meaning units, condensation, and codes. To limit the number of codes, without losing accuracy, a record of codes was available to all authors. The already existing codes could be re-used, if possible, but also new ones were continuously added. In total, 405 codes were settled on. As the work resulted in many codes, these were sorted into content areas by two of the authors (IL, CL). After this, all the authors gathered on several occasions for joint discussions on how to group and sort codes into subcategories and categories in a process that went back and forth. During the whole analytic process, the researchers worked close to the text. Finally, three main categories with eight sub-categories were settled on. We also identified an overall theme that was present throughout the complete material. The theme included text that was not possible to categorize, and impacted all categories. To add transparency and trustworthiness [30] to the findings, quotations are added to the results. The analysis process is illustrated in Table 2.

**Table 2:** Examples illustrating the coding tree

Meaning unit *(citation)*	Condensed meaning unit	Code	Subcategory	Category	Theme
*And this morning when I woke up I thought” Oh no; think if the pandemic ends, then I´ll have to be at the workplace maybe five days a week and that I don´t want. “So, it went from “ Oh God no!” to “ I wish this carries on”. [Participant 3]*	Do not want to be five days/week at the workplace after the pandemic	Ability to work from home was preferred and gave advantages	Ability to work from home led to increased efficiency and more flexibility in daily life	New possibilities of flexibility in work and better health	Both hindrances and opportunities in important life domains were experienced within the same person during lenient Covid-19 restrictions
*…some think that you ought not to go the gym, but if I don´t go I´ll feel pretty low…both in my head and body. I need it, and it works well for me as I can pre-book a time and there are few people there. You´re by yourself at the barbel…it feels safe enough and people keep their distance, so I don´t think that I have to feel guilty about it. [Participant 6]*	Works well with the gym when booking and keeping distance	Physical activity as usual despite restrictions	Physical activities and leisure time continued as before	Life went on as usual with minor adjustments	
*As a matter of fact, most concern is for older people who might not get through an infection, they are the ones we are most worried over, more than normal… That´s the worry we have that somebody we know with poor health might not get through an infection. [Participant 9]*	Worries for older people	New worries caused by the pandemic and restrictions	Increased worries for others and the future	Everyday life changed and became more difficult	

## Results

The 15 participants’ median age was 49 (range 26–76) years, seven were women. They were equally distributed among the four age groups. The size of residential community areas was also evenly distributed between the participants, where five lived in large cities, six in medium- sized cities and four in villages. Four participants had children living at home part time or full time. Regarding income, eleven were working, one was studying and three responded to the response option “other”. There were differences regarding whether they could work from home during the pandemic or not, where three persons had to be at their workplace. The majority of the participants were born in Sweden (85,7%) [21]. For further information see Table 1.

**Fig. 1:**
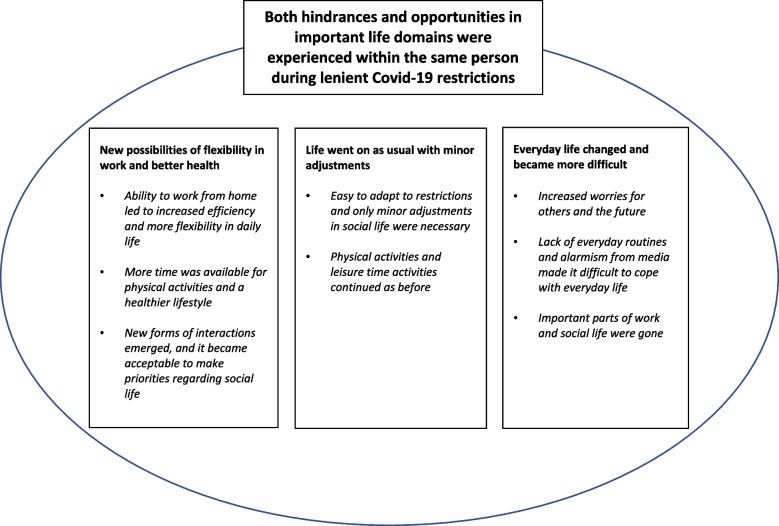
An overview of the theme, categories and subcategories

The results are presented with an overall theme.”Both hindrances and opportunities in important life domains were experienced within the same person during lenient Covid-19 restrictions” and three main categories 1) “New possibilities of flexibility in work and better health”, 2) “Life went on as usual with minor adjustments” and 3) “Everyday life changed and became more difficult”. The first category had three sub-categories, the second had two, and the third three sub-categories (Fig. 1).

### Both hindrances and opportunities in important life domains were experienced within the same person during lenient Covid-19 restrictions

The pandemic and the restrictions were experienced in different ways, very much based on how the participant could tackle the new situation. Personal resources such as mental health and social network impacted how the participant coped with the situation. Also, more general living conditions were important since these could serve as facilitating or hindering factors.

The impact of the pandemic was multifaceted. For most participants, both facilitating and hindering important domains in life were described. Still, prior activities and interests, social networks and working conditions, were important for how the pandemic and the restrictions affected different areas of everyday life. For individuals working, possibilities to organize and perform the work with respect to restrictions, had a great impact.

### New possibilities of flexibility in work and better health

For many of the participants, the challenges following the restrictions also opened opportunities and facilitated changes for the better. To work from home was a new experience for many, which was appreciated. Thereby, more time was available for physical activities and a healthier lifestyle. Also, cancelled activities contributed to a quieter lifestyle. New forms of interactions were experienced and prioritizing in one´s own social life was approved.

#### Ability to work from home led to increased efficiency and more flexibility in daily life

To work from home provided benefits of more flexibility and greater freedom to plan and perform the work. Also, the breaks were more effective, since doing minor home duties during pauses were mentioned. Overall, there was more acceptance from the employer and colleagues for what could be done during the day, for example to take a walk while in a meeting. Also, the technical solutions for social networking developed during the pandemic. Digital meetings were perceived to be more effective than meetings in real life since no time was wasted on commuting. But digital meetings with a creative purpose and with persons with whom one was not well acquainted, were considered difficult. Participants who had that type of work missed having meetings and conferences in real life. Many hoped that they should be able to work partly from home after the pandemic, for example some days a week, since there were vast benefits.

*And this morning when I woke up I thought” Oh no; think if the pandemic ends, then I´ll have to be at the workplace maybe five days a week and that I don´t want. “So, it went from “Oh God no!” to “I wish this carries on”. [Participant 3].*

#### More time was available for physical activities and a healthier lifestyle.

Some participants described their physical health as better than before the pandemic. The pandemic had given opportunities for physical activities through flexible working hours and cancelled activities for both the participants and their children.

Some practiced physical activity on a high level and had increased the time spent on physical activities even further, up to 5–7 times per week. Yoga, gym, cross-country running, padel, badminton, and strength training were examples of strenuous physical activities.

*Yes, I train quite a lot. I train every day…Maybe like 17–18 h a week thereabout…I have actually increased [during the pandemic] (laughs). Due to the lack of other things as well…that´s what you do…Have trained more often and played [badminton]with a few mates I like to play with. [Participant 9].*

#### New forms of interactions emerged, and it became acceptable to make priorities regarding social life

Alternative ways to communicate were found, as using Facetime and Zoom. For some, this was already the case before the pandemic a common way to communicate that could now be extended. But for others it was a new way of keeping in contact with friends and relatives. It was perceived as a convenient way to keep in touch and led for some to have even more contact than before the pandemic.

Changed or less social activities, minimized travelling and changed work circumstances also contributed to a less hectic everyday life. Many expressed that they lived a more peaceful life and had more time on their own. This was appreciated and generated a better balance in life. Moreover, some had reflected on which persons and activities were important in their life and prioritized the time with the close family and a small group of nearest relatives or friends. For these participants, the social arena had become smaller.

Furthermore, many participants also reflected on how good habits could be established after the pandemic. An example mentioned by several, was that reduced travelling could minimize the environmental impact. Even if many longed to travel and meet their friends again, some also hoped to go on with the calmer way of life.

*Yes [on the positive side] I have taken things easier and not stressed as much. I believe that many have been constantly stressed….and we have wound down a little, not all the time having so many different things to do….I haven´t used my work calendar for a year now, no time schedule to keep track of in a way. [Participant 12].*

***…****I don´t think that people will travel as much as before and they´ll appreciate dear Sweden to be a beautiful country that has all we need….we won´t need to fly abroad as much as we did before like…work trips or private trips like. [Participant 11].*

### Life went on as usual with minor adjustments

For many participants, it was considered important to live as “normal” as possible (generally implying “as before the pandemic”). Several participants had a pragmatic view and described themselves as having easy to adjust. They experienced that it was possible to take active decisions within the restrictions and that they only had to make minor adjustments to their lives. Participants with this attitude did not let the restrictions influence life more than necessary and did not find the restrictions burdensome. Also, some lived already in a manner that was not affected by the pandemic.

#### Easy to adapt to restrictions and only minor adjustments in social life were necessary

Many participants accepted the restrictions and considered that they followed them, for example went to the supermarket when it was not crowded or met friends outdoors. Even if some participants would have wished harder restrictions, many were content with the authorities and their management decisions and felt safe. They also expressed that it was positive that there was no lockdown and that it was beneficial for the economy. Moreover, many described good mental and physical health and took responsibility for maintaining good health. They lived their life mainly as before and chose not to ponder upon the negative consequences of the pandemic.

*I think that the authorities made the, the best decisions they could make given the situation, and after that you just had to go along with it. If it was right or wrong in hindsight only time will tell but everyone did as good as they could when we were up in it. [Participant 3].*

*But then again…one is more active…quite a lot…Even with the restrictions, we could actually be there…It felt pretty good to be at home and plan the day just as I liked, especially in the morning with the kids. Same thing if I wanted to meet up with a friend or someone after work. [Participant 13].*

#### Physical activities and leisure time activities continued as before

Physical activities and training were for many an important part of everyday life, and many tried to exercise as before the pandemic. Even if gyms were partly placed under restrictions (i.e., limited number of visitors), they were not completely closed, and the participants tried to participate in the activities that were available.

Many tried to perform physical activities and leisure activities outdoors; examples were walking, running, cycling, skiing or hunting. Other participants had a dog or a horse, which they spent their leisure time with. For those who prior to the pandemic neither had taken part in cultural activities, visited restaurants, nor were used to spending time going shopping, the restrictions did not incur great changes. Neither indoor leisure activities, such as reading, playing computer games, engaging in conversations with relatives and friends via telephone and watching TV were affected. During holiday time, the participants described travelling in Sweden. Even if some thought that not being able to go abroad had a negative impact, not many expressed that they missed it.

*…some think that you ought not to go the gym, but if I don´t go I´ll feel pretty low…both in my head and body. I need it, and it works well for me as I can pre-book a time and there are few people there. You´re by yourself at the barbell… it feels safe enough and people keep their distance, so I don´t think that I have to feel guilty about it. [Participant 6].*

### Everyday life changed and became more difficult

Everyday life could also be perceived as being disturbed during the pandemic. The restrictions in society were for some a heavy burden. Common but unwelcomed effects of the pandemic were an increased feeling of anxiety and lack of everyday routines.

#### Increased worries for others and the future

Worrying was a prominent part of perceptions described. The degree of concerns changed from time-to-time but was in general directed at others, such as relatives and people in their immediate surroundings. The participants expressed being worried for older relatives and their well-being. Some did not visit their relatives, to not expose them to potential infection. Among those, there were sometimes also older adults at risk for developing severe disease. One´s own health was in general described as being of lesser importance when compared to other concerns. But participants working in for example institutions or hospitals had to stay in their workplace where they had to meet clients or patients. They described experiences of higher workload when colleagues were in quarantine and of being uncomfortable regarding their risk of being infected, which triggered worrying.

Sports and leisure activities for children were suspended. The lack of activity for children and teenagers worried parents. Also, some participants expressed concerns for the industrial, commercial and cultural spheres and how restaurants could manage with a considerable loss of guests. They felt sorry for those who had been forced into a troublesome situation.

So much was unknown, and the future was unpredictable. There were thoughts on how prepared we are as a society for new pandemics.

*…. We have had different views on all of this, where I have been much much more restrictive than my partner has been. I worried very, very much and thought “Good Lord we shouldn’t meet anyone” and then, then he heads off and has coffee with a mate and I just think “ Oh no no no”…You just get entrenched in thinking that what you do yourself is right and then someone else has another way of seeing things. [Participant 3].*

*As a matter of fact, most concern is for older people who might not get through an infection, they are the ones we are most worried over, more than normal… That´s the worry we have that somebody we know with poor health might not get through an infection. [Participant 9].*

#### Lack of everyday routines and alarmism from media made it difficult to cope with everyday life

For some, the restrictions rendered feelings of increased loneliness and isolation. When everyday routines were not present anymore, it was sometimes hard to find new ones. Mental health was strained, and already present problems became more obvious. For a participant who had contracted the COVID-19 virus and suffered long-COVID-19, everyday life was severely impacted for a long time, not knowing when it would end.

Some participants pointed out the media as a very negative factor that contributed to alarmism. The frequent reporting from all over the world on the number of deaths due to COVID-19 and the increased number of infected was not supportive. Some participants also expressed being somewhat shocked over how the world reacted during the pandemic; the perceived excessive alarmism was difficult to cope with.

*Most of all it´s the hysteria from the papers and journalists on TV, radio, hum, that they just keep on talking about this negatively, negatively, negatively. Hum, I don´t hear anyone saying anything positive like. [Participant 4].*

#### Important parts of work and social life were gone

Not all participants were positive to changes caused by the pandemic. For some, work was experienced to be worsened, with a higher workload when colleagues were in quarantine due to the restrictions. A student described that different elements of the curiculum, such as professional training, were put on hold and not replaced. Collaboration was digital and this resulted in feelings of being lonelier at home.

Also, some participants described that social activities were negatively affected. They felt it was harder to keep up social relations at distance with digital meetings and social media and missed meeting people in more public areas. Also, engagement in different organizations was strongly diminished when meetings were postponed and activities cancelled.

*..,it´s actually quite fun when you´re working like that in a voluntary organisation and there´s just a few working…It´s a tight group when we meet and do things. I haven´t been able to do that for a year now… and I miss it…you don´t get that bonding when we do things together and can meet and sort of just talk with someone who is newly elected… [Participant 15].*

## Discussion

In the present study, 15 individuals with no underlying disease were interviewed regarding perceptions of everyday life with lenient restrictions during the first wave of the COVID-19 pandemic in Sweden. We found that both hindrances and opportunities in important life domains were experienced within the same person during lenient Covid-19 restrictions.

### New opportunities

The most unexpected result in the present study was that almost all participants described positive changes and new possibilities in everyday life during the pandemic and the subsequent restrictions. Although many previous studies of everyday life in connection to COVID-19 describe negative perceptions, also positive experiences can be found [11, 12, 31]. For example, increased social support and reduced loneliness were described and were suggested to increase closeness and social cohesion [3]. Experiences of “Being part of an extraordinary experience", "Re-discovering family”, to be in a close relationship even at a distance and opportunities to spend more quality time with the family have also been described by Italian and UK adolescents [31, 32]. Very similar experiences were expressed in the present study, where the participants reflected on which persons and activities were important in their lives, prioritizing the time with the near family and friends, but in a smaller social arena than before.

Working from home was another challenge that was also experienced as facilitating changes to the better in the present study. Working from home entailed more time for physical and other self-elected activities, and the possibility to be able to have control over one’s daily schedule was much appreciated. Similar experiences are described in previous literature where Zalat et al. found that more than half of the participants in their study recommended that, due to the benefits in their working and social life, working from home should continue also post-COVID-19 [33]. One reason for these positive perceptions can be explained by the Karasek’s job-demand control model, hypothesizing that control buffers the impact of job demands [34]. However, also negative perceptions are reported in previous studies in connection to working from home, since for example parents have had to take care of children simultaneously because of closed schools and kindergartens resulting in a decrease in their well-being [35]. No such experiences were described in the present study, possibly due to that schools and pre-school and nursery care in Sweden were kept open during the pandemic.

In issues related to physical activity and healthy life choices, positive experiences of having more time for such activities were described in the present study. These results are in line with previous results of additional physical health and ability to exercise, as related to the lockdown in UK – a country practicing wide restrictions on movement and socialization and lockdowns also for schools [31]. These authors also reported that the restrictions did not allow them to engage in the type of exercises they wanted, creating new exercising opportunities such as “exercise classes on zoom” [31]. However, in contrast to results describing more time for physical activities, lockdowns in Australia and in the United States were reported to be associated with lower levels of physical activity, poorer mental well-being, along with higher levels of loneliness as compared to a post-lockdown period [36, 37]. Also, and in contrast to the results here, a systematic review with data from 35 countries, presented that the impact of lockdown on movement behaviors showed reduced physical activity and that stricter lockdowns tended to have a larger impact on detrimental movement behaviors [38]. Since more extensive restrictions have been associated with more symptoms of anxiety and depression as compared to social distancing [37], it cannot be excluded that the positive experiences of for example health and more time for physical activity described here may be due to the more lenient restrictions. Open fitness centers, shops, and theatres with restricted number of visitors were implemented and practiced in Sweden, as compared to countries practicing more extensive restrictions. But also the circumstance that Swedish citizens before the pandemic had a high standard of living, well developed digitalization and health care but also a generally high confidence in authorities, can be speculated on to be related to the experiences of opportunities and less strong negative perceptions during the pandemic. Still, the result of this study showing increased physical activity, is not in line with the results from Public Health Agency in Sweden (FHM), describing decreased physical activity [8]. The reason for these differences might be that the population studied here were older (mean age 50 years), since FHM concluded that young people’s lifestyles had changed the most.

### Divergent perceptions of how everyday life was perceived

Participants in the present study described good mental and physical health and took responsibility for maintaining good health. For some participants, work, physical activity, and leisure time activities were affected only to a minor extent. Previous literature mainly describes challenges in relation to the COVID-19 pandemic with regard to physical and mental health. In these studies, challenges in connection to stress, sleep, loss of routines, challenges in connection to others and to employment and finances are emphasized [11, 12, 31, 39].

In the present study, increased anxiety or worry was mainly described as being directed towards others, such as relatives and the surrounding world. For example, several participants expressed worries for older relatives and their well-being, but less so regarding one’s own health. Also, worrying about not exposing others to potential infection and severe illness was expressed by several participants, and was described as a main reason to follow recommendations and restrictions. This resulted in some participants not visiting their older relatives. The future was also in focus for worries along with issues about interpreting the restrictions differently. This is in line with other studies of the pandemic, describing anguish related to death and loss as the most representative topics, and that several emotional and psychological conditions including fear, anxiety, depression and suicide ideation can be triggered by the pandemic itself, but also by the preventive measures taken [11, 32].

Other areas of concern during the pandemic described as a major problem in prior studies have been employment and financial issues [31]. This was not brought up as a major concern by the participants here, but some of them expressed worries for those who had been forced into a troublesome situation because of losing work or worries for the industrial and commercial spheres, cultural life and how restaurants would manage financially. For some of the participants, work was perceived as being worsened with a higher workload when colleagues were in quarantine. A higher workload causing feelings of stress and depressive symptoms are frequently described in previous literature in relation to health care professionals during the pandemic [40, 41].

In the present study, feelings of increased loneliness and isolation were expressed. When everyday routines were not present anymore, and important aspects of work and social life were absent, previous present problems were even more difficult to cope with. Such experiences are in line with previous results of a rapid review of 24 studies from 13 different countries around the world. They concluded inconsistent results, but that it was clear that loneliness had had an impact on mental health of adults worldwide [42]. Interestingly, Benke et al. found in a cross-sectional online survey of 4335 individuals in Germany, that a higher level of restrictions due to lockdown measures, was associated with more loneliness, higher psychosocial distress, and lower life-satisfaction, but not to anxiety and depressive symptomatology [4]. The results by Benke et al. gives us further confidence to believe that although feelings of increased loneliness and isolation were expressed by the present study’s participants, the many concurrent positive experiences expressed, might be due to the lower level of restrictions practiced in Sweden.

The more lenient Swedish restrictions in response to COVID-19 have been described as implying “mild law and individual responsibility”. This means that no lockdowns were implemented, no penalties were issued for violation of restrictions, no schools for minor children were closed [7] as compared to for example Italy’s or UK’s “tough” response to the pandemic [31, 43]. In Sweden, voluntary restrictions on distancing, working from home and for example a maximum number of customers/spectators in shops and theaters were suggested instead. This implicates that the individual had to make a judgement on how to act and make choices in everyday life. Personal judgements could be perceived as troublesome, for example when elderly people did not follow the restrictions and thereby were at risk of being infected, creating tensions between older and younger generations. However, the present study indicates that freedom of decision was appreciated and perceived as positive. It is important to notice that a diversity of experiences was included within the same person, which can be related to the possibilities to adapt in important life domains.

### Strengths and limitations of the present study

The present study encompasses 15 participants, which can be considered as a reasonable sample for an in-depth interview study, to be able to overview the transcribed interviews and provide with an in-depth analysis. We also found that little further information was added during the last interviews. All accepted to participate on first request. The sample is strategic to provide a diversity regarding age, sex, living area and children living at home or not.

The original sample is of a large population that answered a survey on Facebook, meaning that the sample is a selection of individuals using this social media. We have experienced from previous studies that the most satisfied patients, and perhaps also individuals with no disease, may be the most likely to respond to surveys [44]. This might be one of the reasons for the unexpected result describing several positive experiences and opportunities during the pandemic. If the sample had consisted of participants that had lost relatives, work, or income during the pandemic instead, they probably would have responded differently. This should be considered when the results are interpreted. Yet, since the participants were interviewed on current perceptions, no recall bias was present. Even if recruitment from Facebook may lead to selection bias, we believe that our strategic sample from the interviews can give important information of how people experienced the pandemic and lenient restrictions in Sweden.

Another aspect to consider is that the interviews were performed by telephone or on Zoom and not in real life. This might have both negative and positive implications since the interviewer did not see gestures and facial expressions and therefore took no such field notes. Still, several participants described that the digital situation was relaxed and that they felt that they could express their perceptions freely. Nevertheless, interviewing in real life was not possible during the pandemic. The interviews lasted for 21–54 min (mean 39 min). To obtain elaborated answers and a rich material we addressed different topics with open-ended questions using “how”, “please tell me about…”.

In the present study, all authors were engaged in both study-design, in interviewing, coding, formulating sub-categories, categories and a theme and in writing the manuscript. Concerning reflexivity, all researchers were women. Also, all the researchers had to follow the same Covid restrictions as the participants, for example work from home and limit social activities. In some of the interviews, or part of the interviews, the researcher’s experience might have been the same as the participants, but the researchers as well as the participants had a variety of work situations and social situations and were of different ages. The participants were both men and women. To overcome the issues, we therefore continually discussed the results during the analysis, this in order to remain neutral and to eliminate possible influences, which might be brought about by our preunderstanding [45].

Also, we used the COREQ checklist as a foundation when reporting the results [23]. This ensures that several aspects of the interviews and several opinions have been discussed and considered when processing the results and should be regarded as a strength of the study.

## Conclusions

The impact of the pandemic and lenient restrictions in Sweden on the participants´ everyday life was multifaceted. Both hindrances and opportunities in important life domains were experienced within the same person. An increased flexibility in work- and leisure activities were perceived having positive effects for health and wellbeing and led to a better balance in life.

### Supplementary Information

**Additional file 1. **

## Data Availability

The data generated and/or analyzed during the current study are not publicly available. They are available (in Swedish) from the corresponding author on reasonable request, subject to approval from the ethics committee that approved the study.

## Abbreviations

WHO: World Health Organisation
TV: Television
UK: United Kingdom
COREQ: COnsolidated criteria for REporting Qualitative research
FHM: Folkhälsomyndigheten, Public Health Agency in Sweden
