# Supplementary material for: Perceptions of everyday life during lenient COVID-19 restrictions in Sweden- an interview study
Source: BMC Public Health. 2023 Sep 7;23:1743. doi: 10.1186/s12889-023-16599-3 (PMC10483720; doi:10.1186/s12889-023-16599-3)
Supplement: Supplementary file 1 — Additional file 1. [file 12889_2023_16599_MOESM1_ESM.pdf]

Interview guide

We would like to talk to you about your perceptions and experiences during the pandemic and restrictions

-Tell me a little about yourself!

-How does your life situation look like today?

-In what way have your life situation changed during the pandemic and restrictions?

-How does your work situation look like today?

-How would you describe your physical wellbeing today?

-How would you describe your mental wellbeing today? -In what way has your wellbeing changed during the pandemic and restrictions?

-Tell me, how do you maintain social contacts?

-How have you been able to carry out leisure time activities and everyday activities during the pandemic and restrictions?

-How physically active are you today? Is this a change from before? In which way has it changed since before the pandemic?

-In a situation like this, some persons may be worried, while others might think there is no problem in handling the situation. What is your experience?

-Can you tell me about any positive experience during the pandemic and restrictions? How can you use this experience?

-Can you tell me about any negative experience during the pandemic and restrictions? How can you use this experience?

-When you think about the year that has passed during the pandemic and restrictions, what are your thoughts? How have your thoughts varied over the year?

-What are the most important experiences from this year?

-Is there anything else you would like to add?
